# Identification and Localization of Endotracheal Tube on Chest Radiographs Using a Cascaded Convolutional Neural Network Approach

**DOI:** 10.1007/s10278-021-00463-0

**Published:** 2021-05-23

**Authors:** Su Kara, Jake Y. Akers, Peter D. Chang

**Affiliations:** 1 Capistrano Valley High School, Mission Viejo, CA 92692 USA; 2 University of California, Irvine, CA 92697 USA

**Keywords:** Deep learning, Convolutional neural network (CNN), Endotracheal tube (ETT), Chest radiograph (CXR), Carina, MIMIC-CXR

## Abstract

Rapid and accurate assessment of endotracheal tube (ETT) location is essential in the intensive care unit (ICU) setting, where timely identification of a mispositioned support device may prevent significant patient morbidity and mortality. This study proposes a series of deep learning-based algorithms which together iteratively identify and localize the position of an ETT relative to the carina on chest radiographs. Using the open-source MIMIC Chest X-Ray (MIMIC-CXR) dataset, a total of 16,000 patients were identified (8000 patients with an ETT and 8000 patients without an ETT). Three different convolutional neural network (CNN) algorithms were created. First, a regression loss function CNN was trained to estimate the coordinate location of the carina, which was then used to crop the original radiograph to the distal trachea and proximal bronchi. Second, a classifier CNN was trained using the cropped inputs to determine the presence or absence of an ETT. Finally, for radiographs containing an ETT, a third regression CNN was trained to both refine the coordinate location of the carina and identify the location of the distal ETT tip. Model accuracy was assessed by comparing the absolute distance of prediction and ground-truth coordinates as well as CNN predictions relative to measurements documented in original radiologic reports. Upon five-fold cross validation, binary classification for the presence or absence of ETT demonstrated an accuracy, sensitivity, specificity, PPV, NPV, and AUC of 97.14%, 97.37%, 96.89%, 97.12%, 97.15%, and 99.58% respectively. CNN predicted coordinate location of the carina, and distal ETT tip was estimated within a median error of 0.46 cm and 0.60 cm from ground-truth annotations respectively. Overall final CNN assessment of distance between the carina and distal ETT tip was predicted within a median error of 0.60 cm from manual ground-truth annotations, and a median error of 0.66 cm from measurements documented in the original radiology reports. A serial cascaded CNN approach demonstrates high accuracy for both identification and localization of ETT tip and carina on chest radiographs. High performance of the proposed multi-step strategy is in part related to iterative refinement of coordinate localization as well as explicit image cropping which focuses algorithm attention to key anatomic regions of interest.

## Introduction

**Fig. 1:**
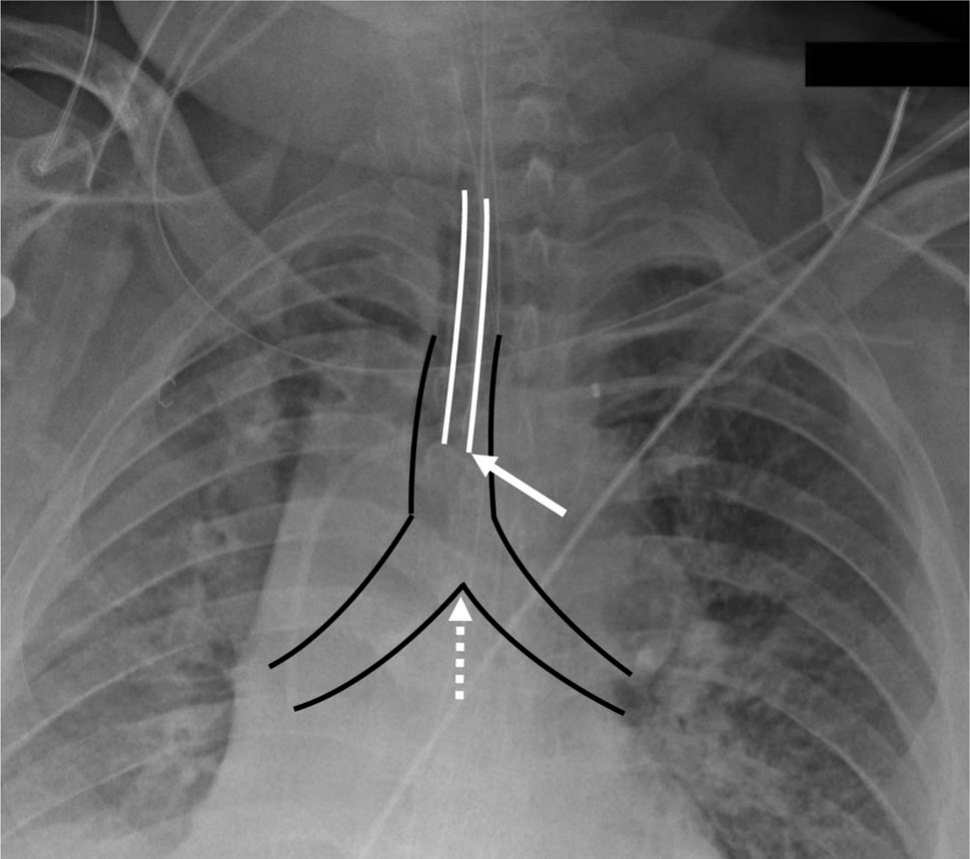
Frontal chest radiograph Single frontal radiograph demonstrating position of an endotracheal tube (ETT; solid white outline) relative to the trachea and proximal right and left bronchi (solid black outline). Optimal positioning is commonly evaluated by characterizing the distal ETT tip (solid white arrow) relative to the carina (dotted white arrow)

An endotracheal tube (ETT) is a plastic airway device placed through the mouth into the trachea and connected to a breathing device to provide mechanical ventilation to the lungs. (Fig. 1) [1]. Optimal positioning of an ETT is essential for therapeutic effect and is commonly cited to be approximately 5 ± 2 cm above the carina [2]. By contrast, a misplaced ETT may often result in serious complications including the collapse or hyperinflation of a lung [3], the overall risk of which may be as high as 40% in the ICU setting [4]. ETT position is typically assessed using a frontal chest radiograph. However, due to high clinical volume, the delay in average turnaround time (TAT) for emergent chest radiographs is approximately 22.89 min [5]. Thus, an objective and accurate tool for characterization of ETT location on chest radiographs would be an invaluable asset in the acute care setting.

In this study, we propose a series of deep learning-based algorithms for identification and localization of ETT position relative to the carina from chest radiographs. Deep learning convolutional neural networks (CNN) are a specific form of data-driven machine learning with the capacity for self-organized feature extraction. Compared to traditional machine learning techniques, supervised deep learning approaches can learn relevant, high-order patterns from data in an end-to-end manner, requiring only raw input and target output pairs of data. In recent years, CNN-based techniques have been recognized as state-of-the-art on various medical imaging tasks related to chest radiograph analysis, including pneumonia detection [6] and characterization of tuberculosis [7].

Given the complexity of the problem, the proposed algorithm is separated into three components in part analogous to steps required by a human radiologist to perform a similar task: (1) coarse localization of the carina (used to focus the algorithm on relevant anatomic regions of interest), (2) determination of the presence or absence of an ETT, and (3) localization of the ETT tip relative to the carina. Each individual component of this algorithm is implemented via an independently trained CNN. Through this serial cascade of deep learning networks, we hypothesize that the iterative refinement in model predictions will allow for characterization of ETT position with high speed and accuracy.

## Materials and Methods

### Data

The MIMIC-CXR Database v2.0.0 is an open-source critical care cohort of chest radiographs in DICOM (Digital Imaging and Communications in Medicine) format with free-text radiology reports and was used for analysis in this project [8–10]. The full dataset contains 377,110 images corresponding to 227,835 reports of 65,389 patients performed at the Beth Israel Deaconess Medical Center in Boston, MA, between 2011 and 2016.

Using a natural language processing (NLP) technique based on regular expressions, all patient reports from the full dataset were parsed to identify the presence or absence of an ETT. From the initial cohort of 227,835 studies, a subset of 8000 patients with an ETT and another 8000 patients without an ETT were identified based on high probability NLP predictions. These 16,000 studies corresponded to 24,010 chest radiographs in the dataset, from which a total of 17,050 frontal radiographs were isolated using DICOM headers. This final dataset was split into five separate cross-validation folds for training. Importantly, all patients with repeat examinations were placed into identical cross-validation folds.

### Annotation

First, all 17,050 frontal radiographs were visually inspected to confirm the presence or absence of an endotracheal tube as labeled by the NLP algorithm. Second, a randomly chosen subset of 7396 frontal images were manually annotated to identify (1) the coordinate location of the carina and (2) the coordinate location of the distal ETT tip if present. Finally, for all patients with an ETT, the measured distance of the ETT tip relative to the carina as documented in the radiology free-text report was recorded. All annotations were reviewed for accuracy by a board-certified radiologist.

### Image Preprocessing and Augmentation

All images were zero-padded until the original matrix was equal in height and width. Subsequently, the square-padded image was resampled to a uniform matrix size of 512 × 512. All images were normalized independently with a *z-*score transformation by subtracting the image mean and scaling by the image standard deviation.

During the training process, data augmentation was applied dynamically to all images to improve model generalizability. First, the image mean and standard deviation values used for data normalization were randomly scaled between 90 and 110%. Additionally, random image translations and scales were applied to both original image input and ground-truth coordinate points, with values ranging from 0 to 14 pixels in displacement.

### CNN Architecture

**Fig. 2:**
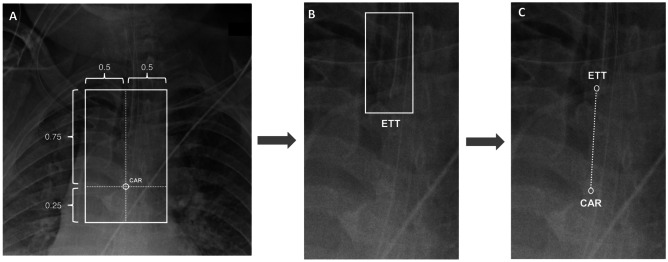
Serial multi-step CNN architecture A total of three different CNN algorithms were created. **A** The first algorithm comprised of a regression loss function network designed to output the estimated (*y*, *x*) coordinate of the carina. From this coordinate position, a 256 × 128 crop of the upper airway was generated. **B** Using this cropped input, a second CNN binary classifier network was used to predict the presence or absence of ETT. **C** Finally, for radiographs containing an ETT, a third CNN regression network was designed to output the estimated (*y*, *x*) coordinates of the carina and the tip of the distal ETT. Abbreviations: CAR carina, ETT endotracheal tube

A total of three different CNN algorithms were created (Fig. 2). The first algorithm comprised of a regression loss function network designed to output the estimated (*y*, *x*) coordinate of the carina. From this information, a 256 × 128 crop of the upper airway was generated. Using this cropped input, a second CNN binary classifier network was used to predict the presence or absence of ETT. Finally, for radiographs containing an ETT, a third CNN regression network was designed to output the estimated (*y*, *x*) coordinates of the carina and the tip of the distal ETT.

**Fig. 3:**
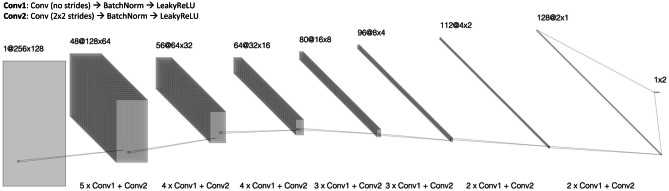
Common CNN backbone For ease of model development, a common CNN backbone architecture was used for all three target tasks, with slight modifications in input matrix shape and loss function between each network. The final network topology comprised a total of 30 convolutional blocks, defined as serial application of a 3 × 3 convolutional operation, batch normalization, and a LeakyReLU activation function. Subsampling was implemented via convolutional operations with a stride of 2. A total of 7 subsampling operations were performed; with each decrease in feature map size the corresponding channel depth was increased from 48 to 128

For ease of model development, a common CNN backbone architecture was used for all three target tasks, with slight modifications in input matrix shape and loss function between each network. Each convolutional operation in the network is implemented using simple blocks, defined as serial application of a 3 × 3 convolutional operation, batch normalization and a LeakyReLU activation function [11]. After a brief architecture hyperparameter sweep, the final network topology comprised a total of 30 convolutional blocks (Fig. 3). Subsampling was implemented via convolutional operations with a stride of 2. A total of 7 subsampling operations were performed; with each decrease in feature map size the corresponding channel depth was increased from 48 to 128.

#### Carina Localization

The first CNN algorithm was designed to predict the *x-* and *y-*coordinates of the carina on all frontal chest radiographs. The model input was the original squared-padded 512 × 512 resampled raw radiograph image. The network was designed with a regression type loss function using mean squared error (MSE) to minimize the loss between the final logit score and the expected normalized coordinate point.

#### ETT Classification

The second CNN algorithm was designed for binary prediction of the presence or absence of ETT tube. The model input utilized the initial prediction of carina location to create a focused crop of the distal trachea and proximal bronchi. Specifically, this crop was defined as a rectangle with a 2:1 aspect ratio (height-to-width) spanning 50% and 25% of the original radiograph, respectively, and centered around the carina as shown in Fig. 2. After cropping, the image is resampled to a uniform 256 × 128 matrix shape. Using this input, the network was trained with a standard binary softmax cross entropy loss function.

#### Carina and ETT Localization

The third CNN algorithm was designed both to predict the *x*- and *y*-coordinates of the endotracheal tube and to refine the existing prediction of the carina. The model input was the same cropped 256 × 128 image centered along the carina as in the second CNN algorithm. The network was designed with a regression type loss function using mean squared error (MSE) to minimize the loss between the final logit score and the expected normalized coordinate points.

### Implementation

Training was implemented from random weights initialized using the Xavier uniform distribution [12]. Optimization was implemented via the Adam method [13] with a learning rate of 0.0005 and a batch size of 12. Approximately 4 epochs were required for algorithm convergence for each experiment. After training the model with the learning rate of 0.0005 for 3 epochs, learning rate was divided by 10 and the model was trained for one more epoch to fine-tune the weights.

For all development purposes throughout this study, Google CoLab RRID:SCR_018009 was used with a Python Version 3.6 RRID:SCR_008394 runtime type and a GPU hardware accelerator. TensorFlow [14] Version 2.2.0 RRID:SCR_016345, an open-source software library, was used to develop the CNN algorithms.

### Statistics

A five-fold cross validation technique was used to estimate model performance. For all regression tasks, algorithm performance was reported using both mean and median absolute distance between predicted and ground-truth coordinates in real-world distances (cm). In addition, the interquartile range (25th and 75th percentile predictions) for prediction accuracy was calculated. For the binary classification task, overall accuracy as well as sensitivity, specificity, positive predictive value (PPV), negative predictive value (NPV), and area under the receiver operating characteristic curve (AUROC) were calculated. In addition, the 95% confidence interval for each of these metrics was determined.

## Results

From the original 17,050 frontal radiographs, a total of 8848 ETT positive and 8202 ETT negative images were identified and used in this study. Based on NLP analysis of the radiology reports, the mean, median, 25th percentile, and 75th percentile of the distal ETT tip from the carina were 4.29 cm, 4.10 cm, 3.00 cm, and 5.30, cm respectively.

### Carina Localization

**Fig. 4:**
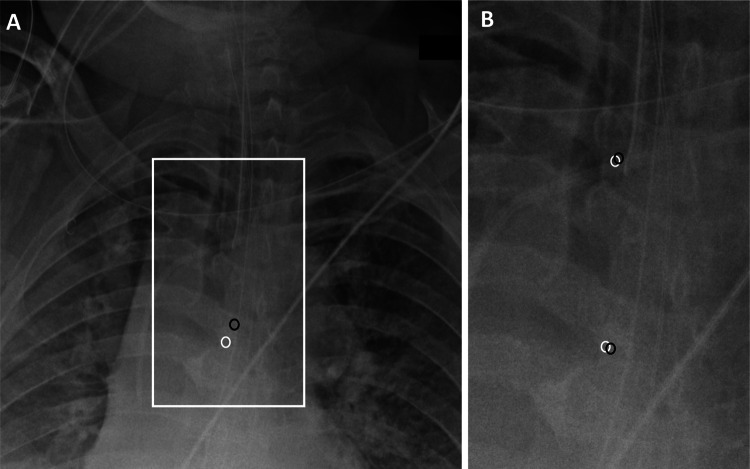
CNN model predictions Estimate of carina location by initial first-step CNN algorithm (**A**), which is subsequently refined by the final CNN algorithm (**B**). In addition, the final CNN algorithm produces an estimate of the coordinate location of the distal ETT. Manually annotated positions are shown as the white circles, while the CNN-derived estimates are shown as the black circles

After aggregating cross-validation cohort results, the mean, median, 25th and 75th percentile absolute errors in prediction of carina location were 1.33 cm, 1.10 cm, 0.67 cm, and 1.68 cm, respectively (Table 1; Fig. 4A). Note that this initial coarse prediction is used primarily as a preprocessing step to localize the attention of the algorithm to the relevant portions of the image. Final prediction of carina location is performed in subsequent steps. Overall inference time for the full cohort was approximately 0.025 s per exam.

**Table 1:** Cumulative performance metrics for CNN localization tasks reported using absolute distance errors (cm)

Task	Mean	Median	25th percentile	75th percentile
Carina coordinate (initial)	1.33	1.10	0.67	1.68
Carina coordinate (final)	0.64	0.46	0.27	0.77
ETT coordinate (final)	0.82	0.60	0.35	1.01
Carina-ETT distance (annotations)	0.86	0.60	0.28	1.14
Carina-ETT distance (radiology reports)	0.86	0.66	0.31	1.16

### ETT Classification

After aggregating cross-validation cohort results, the overall accuracy, sensitivity, specificity, PPV, NPV, and AUROC of the algorithm for differentiating the presence or absence of ETT were 97.14%, 97.37%, 96.89%, 97.12%, 97.15%, and 99.58%, respectively (Table 2). Overall inference time for the full cohort was approximately 0.005 s per exam.

**Table 2:** Cumulative performance metrics for CNN binary classification

Statistic	Value	95% Confidence interval
Accuracy	97.14%	96.88 to 97.38%
Sensitivity	97.37%	97.01 to 97.69%
Specificity	96.89%	96.49 to 97.26%
Positive predictive value	97.12%	96.77 to 97.44%
Negative predictive value	97.15%	96.78 to 97.48%
AUROC	99.58%	99.48 to 99.68%

### Carina and ETT Localization

After aggregating cross-validation cohort results, the mean, median, 25th, and 75th percentile absolute errors in prediction of carina location was 0.64 cm, 0.46 cm, 0.27 cm, and 0.77 cm, respectively. The mean, median, 25th, and 75th percentile absolute errors in prediction of distal ETT tip were 0.82 cm, 0.60 cm, 0.35 cm, and 1.01 cm, respectively (Table 1; Fig. 4B). Overall inference time for the full cohort was approximately 0.015 s per exam.

The final outputs generated by the third CNN algorithm were used to estimate the position of the ETT tip relative to the carina. Using manually annotated ground-truth annotations, the mean, median, 25th, and 75th percentile absolute errors in prediction of ETT to carina distance were 0.86 cm, 0.60 cm, 0.28 cm, and 1.14 cm, respectively. In addition, the CNN-derived estimate of ETT to carina distance was compared to measurements documented in the original MIMIC-CXR radiology report. In this analysis, the mean, median, 25th and 75th percentile absolute errors in prediction were 0.86 cm, 0.66 cm, 0.31 cm, and 1.16 cm, respectively (Table 1).

## Discussion

A serial cascaded CNN approach trained on 17,050 patient images demonstrates high accuracy for localization and assessment of ETT position. Compared to a global binary classifier which may simply predict adequate or inadequate positioning, the proposed deep learning algorithm can provide explicit feedback regarding the exact position of the ETT relative to the carina, helping guide clinical decision making. Based on evaluation of both ground-truth manual annotations as well as radiology reports, the CNN algorithm can generate automated and accurate predictions within 6 mm of expected location in less than one second.

Previous works have evaluated the deep learning in chest radiographs [15] including in the diagnosis of pneumonia [16], tuberculosis [17], thoracic diseases [18], and lung abnormalities [19]. More recently, several works have applied deep learning techniques to characterization of ETTs. In an initial study, Lakhani et al. [20] utilized a global CNN classifier for binary assessment of ETT position without explicit localization. Frid-Adar et al. [21] localizes the entire course of the ETT using a fully-convolutional CNN model for semantic segmentation trained with a combination of both real and synthetic data, however does not characterize the location of the distal ETT tip relative to the carina. Finally, Huo et al. [22] uses traditional computer vision techniques to assess ETT position with a sensitivity of 0.85 for ETT detection and an accuracy of 0.81 for ETT localization within 10 mm of ground-truth annotations. This current work, leveraging an approximate 15-fold increase in data and end-to-end deep learning techniques, yields improved performance metrics with over 0.97 sensitivity for ETT detection and a median error of 6 mm from ground-truth annotations.

Compared to previous efforts which primarily use global classification and/or segmentation approaches, this study uses a landmark-based coordinate regression strategy for carina and ETT localization. Landmark-based coordinate regression algorithms have been successfully implemented in various radiology tasks. Noothout et al. [23] and Tan et al. [24] utilized this technique for localization of key cardiac and vascular anatomy on CT angiography. Theriault-Lauzier et al. [25] followed a similar approach to infer the location and orientation of the aortic valve annular plane. Ma et al. [26] leveraged a coordinate regression method for characterization of the carotid artery bifurcation anatomy. In this work, we extend the use of landmark-based regression to implement both a hard-attention mechanism as well as support device localization.

The serial cascaded CNN approach, in part inspired by the attention mechanism of a human radiologist, allows the algorithm to focus on the relevant portions of the image after first localizing key anatomic regions of interest. This iterative reduction in search space yields significant improvements in algorithm performance, with a median absolute error difference in carina localization of 1.10 cm compared to 0.46 cm before and after image cropping. In addition, focusing the algorithm on a specific sub-region of the image allows for analysis at a relatively high-resolution in a memory-efficient manner. In fact, even though the second and third CNN input is smaller in matrix size than the first CNN input, the real-world size of each individual pixel was smaller (e.g., higher resolution) in the later models.

Several parameter combinations were tested to optimize the common CNN network backbone. After a hyperparameter sweep, optimal permutations tended to favor relatively increased modeling capacity in the initial few layers of the network as implemented through more convolutional layers and increased feature map depth. This observation can likely be explained by the need for fine-detail, high-frequency features to accurately identify small structures such as the carina and an ETT on chest radiographs. Such features can only be learned in the earliest layers of the network topology, prior to significant feature map subsampling. In our top-performing model, a total of 5 convolutional blocks were used before the first subsample, and a total of 4 convolutional blocks were used before the second subsample. By contrast, most top-performing networks in non-medical imaging domains such as the ImageNet Challenge perform aggressive subsampling in the initial CNN layers and instead retain the majority of modeling capacity in the deepest convolutional feature maps. Additionally, the incremental benefit of data augmentation for regression tasks was observed to be approximately 25% across various experiments, a value higher than comparable classification tasks in part related to the improved sampling of latent space afforded by data augmentation for the continuous-variable output regression tasks.

Overall algorithm performance was higher for the task of carina localization (within a median error of 0.46 cm) compared to ETT localization (within a median error of 0.60 cm). We suspect that this difference is primarily due to the inherent difficulty of identifying these two structures. The carina, while subtle in appearance, is identified in a reliable location with consistent anatomic landmarks across most patients. An ETT by contrast may exist in varied locations and may often be confused with other support devices that demonstrate similar course and/or density. In future studies, we anticipate that the performance of ETT localization will be improved with more training data that may better reflect the heterogeneity of possible appearances and locations.

There are several limitations acknowledged by the current study design. While a large number of exams were used to train the proposed deep learning models, the current cohort of radiographs were derived from a single institution. As such, further evaluation is needed to assess performance across a wide variety of manufacturers and image protocols. Additionally, the current algorithm is designed to evaluate frontal chest radiographs only, while in practice a chest radiograph exam may in fact contain alternative projections. While this is relatively infrequent in the ICU setting, and may sometimes be sorted using DICOM headers, a more robust strategy to identify relevant model inputs will be needed before such a tool can be fully integrated into the clinical workflow.

## Conclusions

Rapid and accurate assessment of ETT location is critical, where timely identification of a mispositioned support device may prevent significant patient morbidity and mortality. A serial cascaded CNN approach demonstrates high accuracy for both identification and localization of ETT tip and carina on chest radiographs. High performance of the proposed multi-step strategy is in part related to iterative refinement of coordinate localization as well as explicit image cropping which focuses algorithm attention to key anatomic regions of interest. Future work includes ongoing efforts at our institution for clinical integration of the deep learning tool, including optimization of user interface to minimize workflow disruption and improve overall clinical response time.
